# Linear Discriminant Analysis-Based Motion Classification Using Distributed Micro-Doppler Radars with Limited Backhaul

**DOI:** 10.3390/s21092924

**Published:** 2021-04-21

**Authors:** Yonggi Hong, Yunji Yang, Jaehyun Park

**Affiliations:** Division of Smart Robot Convergence and Application Engineering, Department of Electronic Engineering, Pukyong National University, Busan 48515, Korea; yongki0503@hanmail.net (Y.H.); yyj26@pukyong.ac.kr (Y.Y.)

**Keywords:** linear discriminant analysis, micro-Doppler radar, pyramid vector quantization

## Abstract

In this paper, we propose a cooperative linear discriminant analysis (LDA)-based motion classification algorithm for distributed micro-Doppler (MD) radars which are connected to a data fusion center through the limited backhaul. Due to the limited backhaul, each radar cannot report the high-dimensional data of a multi-aspect angle MD signature to the fusion center. Instead, at each radar, the dimensionality of the MD signature is reduced by using the LDA algorithm and the dimensionally-reduced MD signature can be collected at the data fusion center. To further reduce the burden of backhaul, we also propose the softmax processing method in which the distances of the sensed MD signatures from the centers of clusters for all motion candidates are computed at each radar. The output of the softmax process at each radar is quantized through the pyramid vector quantization with a finite number of bits and is reported to the data fusion center. To improve the classification performance at the fusion center, the channel resources of the backhaul are adaptively allocated based on the classification separability at each radar. The proposed classification performance was assessed with synthetic simulation data as well as experimental data measured through the USRP-based MD radar.

## 1. Introduction

Recently, micro-Doppler (MD) radars have been deployed to detect or recognize target motion due to their low implementation cost and robustness to harsh environmental conditions [1,2,3,4]. Specifically, the target motions such as rotations and vibrations cause small-scale frequency shifts of a radar echo signal, which represent spectrograms that generally differ according to different target motions, also known as MD signatures. Furthermore, MD signatures caused by target motion can be captured through the MD radar whose operation is not affected by the surrounding conditions (e.g., bad weather or light intensity). Accordingly, MD radar-based motion detection and recognition are applied to UAV identification systems [5], human activity classification and smart homes [1,2,3]. However, the MD signature is generated based on the radar echo signal from unstable scattering points of the target and heavily dependent on the aspect angle of the target relative to the radar’s line of sight [6].

To overcome it, MD signatures for multiple perspectives are collected by using multiple radar nodes or a single monostatic radar that traverses the target [7]. In Özcan et al. [8], it is verified that the distributed radar configuration outperforms the colocated MIMO radar configuration because the former obtains multi-aspect MD signature data. However, in open literature addressing the distributed radar configuration for motion recognition [9,10], the backhaul link for distributed radar nodes to share their sensing data (i.e., radar echo signals) is assumed to be wired and have an ideal unlimited capacity, which hinders flexible deployment of the distributed radar system.

In this paper, to get multi-aspect MD signature data, a distributed MD radar system with limited backhaul link is considered, where the distributed radars cannot report the high-dimensional data of MD signature to the fusion center due to the limited backhaul link. Note that the dimension of the MD signature depends on the size of short-time Fourier transform (STFT) and the observation time duration (for example, in our experiment, 64 point STFT was exploited and 2534 time samples were observed for one MD signature; then, the dimensions of the MD signature were $1.62\times 10^{5}$, which could cause the significant overhead to the typical limited backhaul link). Accordingly, for the distributed radar, the dimensions of the MD signature were reduced by exploiting the generalized singular value decomposition (GSVD)-based linear discriminant analysis (LDA) algorithm, which has been successfully exploited in the dimension reduction related applications [11,12,13,14]. Then, we developed the motion classification algorithm by exploiting the dimensionally-reduced MD signature for multiple perspectives collected at the data fusion center. Even though the dimensions of the MD signature data were reduced, a naive element-wise quantization of the dimensionally-reduced MD signature data incurred significant distortion. Accordingly, rather than transmitting the dimensionally-reduced MD signature data, we propose the softmax processing method in which the distances of sensed MD signature from the centers of clusters for all motion candidates are computed at each radar, and the distance can be expressed as a probability through the softmax function. Then, the output of the softmax process at each radar is quantized through the pyramid vector quantization (PVQ) with a finite number of bits and is reported to the data fusion center. We note that PVQ was developed for the data compression and quantization of Laplacian-like data with arbitrary vector dimensions [15,16]. In addition, PVQ is an efficient quantization method when the sum of vector components is fixed as a constant, where the codebook of PVQ consists of cubic lattice points on the *L*-dimensional pyramid. Accordingly, by exploiting the normalizing the constant, we exploit the PVQ to quantize the output of the softmax process. To improve the classification performance at the fusion center, the channel resources of the backhaul link are adaptively allocated based on the classification separability at each radar. Specifically, by allocating more bits (i.e., more resources of backhaul link) to the distributed radar with larger separability, the motion classification performance at the data fusion center can be further improved. The proposed classification performance was assessed with synthetic simulation data (MNIST hand writing data [17]) as well as experimental data measured through multiple MD radars that we implement by exploiting the USRP N210 devices with CBX daughterboards [18].

The rest of this paper is organized as follows. In Section 2, we introduce the system model for a distributed MD radar system and the associated signal model. In Section 3, MD signature-based motion classification using GSVD/LDA is developed for a single radar. In Section 4, we discuss the motion classification at the data fusion center by exploiting the dimensionally-reduced data reported from the distributed radars. In addition, we also propose the softmax processing method to further reduce the burden of backhaul linking and discuss the application of PVQ to the output of the softmax process at each radar. In Section 5, we provide several simulation results and experimental results. In Section 6, we give our conclusions.

## 2. System Model

**Figure 1:**
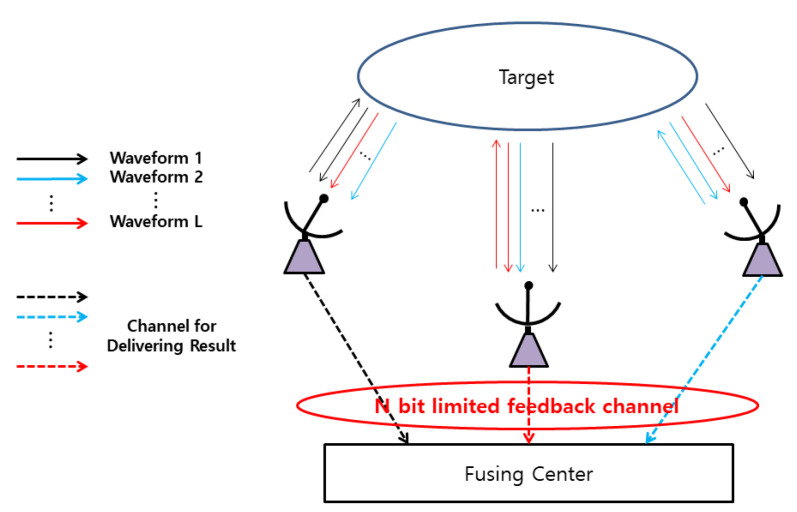
Model of a distributed micro-Doppler radar system.

Figure 1 shows the distributed MD radar system with limited backhaul link, where *L* distributed radars transmit continuous waveforms with different center frequencies. Since the radars are spatially distributed, for the same target motion, each radar may receive the echo signal conveying different MD signature.

When considering *D* target motions to be classified, the discrete received signal of the *l*th radar for the *i*th motion (i=1,…,D) can then be expressed as follows:

(1) $$y_{i}^{\left(l\right)}\left[n\right]=\alpha_{i}^{\left(l\right)}\exp\left(j2\pi \left(f_{c}^{\left(l\right)}+\Delta f_{i}^{\left(l\right)}\left(nT_{s}\right)\right)\left(nT_{s}-\tau_{i}^{\left(l\right)}\right)\right)+\nu^{l}\left[n\right]$$

for n=1,…,N, where $T_{s}$ is a sampling time and $\nu^{\left(l\right)}\left[n\right]$ is the zero-mean additive white Gaussian noise having a variance $\sigma_{n}^{2}$. Here, $\alpha_{i}^{\left(l\right)}$ represents the aggregated channel gain, including path-loss and antenna gain, and $\tau^{\left(l\right)}$ is the time delay associated with the range between the target and the *l*th radar. In (1), $\Delta f_{i}^{\left(l\right)}$ is the micro-Doppler shift characterized by the *i*th motion. In general, this MD signature is a function of time and can be clearly observed in time-frequency domain using short-time Fourier transform (STFT) [4]. Accordingly, the STFT of $y_{i}^{\left(l\right)}\left[n\right]$, n=0,…,N−1 can be given as

(2) $$\begin{array}{ccc} Y_{i}^{\left(l\right)}\left[n,k\right] & = & STFT(y_{i}^{\left(l\right)}\left[n\right],n=0,\ldots ,N-1)\\ & = & \alpha_{i}^{\left(l\right)}STFT\left(f_{i}^{\left(l\right)}\left[n\right],n=0,\ldots ,N-1\right)+STFT\left(\nu^{\left(l\right)}\left[n\right],n=0,\ldots ,N-1\right)\\ & = & \alpha_{i}^{\left(l\right)}F_{i}^{\left(l\right)}\left[n,k\right]+N^{\left(l\right)}\left[n,k\right], \end{array}$$

for k=0,…,M−1, where $f_{i}^{\left(l\right)}\left[n\right]=\exp\left(j2\pi \left(f_{c}^{\left(l\right)}+\Delta f_{i}^{\left(l\right)}\left(nT_{s}\right)\right)\left(nT_{s}-\tau_{i}^{\left(l\right)}\right)\right)$, and STFT can be given as

(3) $$S\left[n,k\right]=STFT\left(s\left[n\right],n=0,...,N-1\right)=\sum_{m=0}^{\;M-1}s\left(n+m\right)w\left(m\right)e^{-j2\pi \frac{mk}{M}},$$

where w(m) is the window function with a length of *M*. In (1), $F_{i}^{\left(l\right)}\left[n,k\right]$ is the MD signature observed at the *l*th radar for the *i*th motion excluding the channel gain, and $N^{\left(l\right)}\left[n,k\right]$ is the STFT of the additive white Gaussian noise. To analyze MD signature $Y_{i}^{\left(l\right)}\left[n,k\right]$ for n=0,…,N−1 and k=0,…,M−1, $Y_{i}^{\left(l\right)}\left[n,k\right]$ is vectorized as

(4) $$Y_{i}^{\left(l\right)}=\left[\begin{array}{c} Y_{i}^{\left(l\right)}\left[0,0\right]\\ Y_{i}^{\left(l\right)}\left[0,1\right]\\ \vdots \\ Y_{i}^{\left(l\right)}\left[N-1,M-1\right] \end{array}\right]\in C^{MN\times 1}$$

From (2), $F_{ij}^{\left(l\right)}$ and $N_{ij}^{\left(l\right)}$ can be vectorized in a similar way as (4), and we can have

(5) $$Y_{i}^{\left(l\right)}=\alpha_{i}^{\left(l\right)}F_{ij}^{\left(l\right)}+N_{ij}^{\left(l\right)}.$$

Throughout the paper, it is considered that the received radar signal at the *l*th distributed radar can be preprocessed and reported to the data fusion center through the limited backhaul link with the capacity of $N_{l}$ bits per channel use. That is, $N_{l}$ bits can be transmitted error-freely for one channel use of the *l*th distributed radar. In addition, it is assumed that

(6) $$\sum_{l=1,...,L}N_{l}\leq N_{total}.$$

## 3. MD Signature-Based Motion Recognition Using GSVD/LDA in a Single Radar

LDA has been used for the signal identification/classification dealing with high-dimensional data efficiently [11,12,13], in which a linear transformation matrix $G\in C^{MN\times M_{d}}$ is computed based on given sample dataset to reduce the dimension of the high-dimensional dataset and simultaneously maximize the separability along different classes, where $D-1\leq M_{d}\ll MN$.

For the motion recognition in the *l*th radar, the MD datasets are collected for each motion. Specifically, by referring to (4), let us denote $Y_{ij}^{\left(l\right)}\in C^{MN\times 1}$ as the *j*th sample for the *i*th motion’s MD signature data. Then, the collected datasets can be formulated as

(7) $$A^{\left(l\right)}=\left[A_{1}^{\left(l\right)},A_{2}^{\left(l\right)},\ldots ,A_{D}^{\left(l\right)}\right]\in R^{MN\times DN_{s}},$$

where $A_{i}^{\left(l\right)}=\left[Y_{i1}^{\left(l\right)},Y_{i2}^{\left(l\right)},\ldots ,Y_{iN_{s}}^{\left(l\right)}\right]$ and $N_{s}$ is the number of samples per cluster (i.e., one target motion). In addition, by using the collected datasets, three scatter matrices are computed as

(8) $$S_{w}^{\left(l\right)}=\sum_{i=1}^{D}\sum_{j=1}^{N_{s}}\left(Y_{ij}^{\left(l\right)}-m_{i}^{\left(l\right)}\right){\left(Y_{ij}^{\left(l\right)}-m_{i}^{\left(l\right)}\right)}^{H}$$

(9) $$S_{b}^{\left(l\right)}=\sum_{i=1}^{D}N_{s}\left(m_{i}^{\left(l\right)}-m_{total}^{\left(l\right)}\right){\left(m_{i}^{\left(l\right)}-m_{total}^{\left(l\right)}\right)}^{H}$$

(10) $$S_{total}^{\left(l\right)}=\sum_{i=1}^{D}\sum_{j=1}^{N_{s}}\left(Y_{ij}^{\left(l\right)}-m_{total}^{\left(l\right)}\right){\left(Y_{ij}^{\left(l\right)}-m_{total}^{\left(l\right)}\right)}^{H},$$

which are respectively called within-cluster, between-cluster, and total scatter matrices [11]. Here, $m_{i}^{\left(l\right)}$ is the average of the samples in the *i*th cluster, given as $m_{i}^{\left(l\right)}=\frac{1}{N_{s}}\sum_{j=1}^{N_{s}}Y_{ij}^{\left(l\right)}$, and $m_{total}^{\left(l\right)}$ is the average of total samples in the collected datasets, given as $m_{total}^{\left(l\right)}=\frac{1}{DN_{s}}\sum_{i=1}^{D}\sum_{j=1}^{N_{s}}Y_{ij}^{\left(l\right)}=\frac{1}{D}\sum_{i=1}^{D}m_{i}^{\left(l\right)}$. Note that the trace of $S_{w}$ implies the variance of sample data within the same cluster, while the trace of $S_{b}$ is the variance of the cluster mean vectors (i.e., $m_{i}^{\left(l\right)}$, i=1,…,D) with respect to $m_{total}^{\left(l\right)}$, providing a measure of the distance between clusters. We note that, at the *l*th radar, by evaluating the Euclidean distance of the received data vector from the representative vectors $m_{i}^{\left(l\right)}$, the motion can be identified thusly:

(11) $$\hat{i}=\underset{i}{\arg\min}{∥Y_{test}^{\left(l\right)}-m_{i}^{\left(l\right)}∥}_{2}^{2}.$$

However, the process dealing with high dimensional data vectors requires high computational complexity.

For the dimensionally-reduced sample data ${\tilde{Y}}_{ij}^{\left(l\right)}=G^{H}Y_{ij}^{\left(l\right)}\in C^{M_{d}\times 1}$, the scatter matrices can be given as

(12) $${\tilde{S}}_{w}^{\left(l\right)}=\sum_{i=1}^{D}\sum_{j=1}^{N_{s}}\left(G^{H}Y_{ij}^{\left(l\right)}-G^{H}m_{i}^{\left(l\right)}\right){\left(G^{H}Y_{ij}^{\left(l\right)}-G^{H}m_{i}^{\left(l\right)}\right)}^{H}=G^{H}S_{w}^{\left(l\right)}G$$

(13) $${\tilde{S}}_{b}^{\left(l\right)}=\sum_{i=1}^{D}N_{s}\left(G^{H}m_{i}^{\left(l\right)}-G^{H}m_{total}^{\left(l\right)}\right){\left(G^{H}m_{i}^{\left(l\right)}-G^{H}m_{total}^{\left(l\right)}\right)}^{H}=G^{H}S_{b}^{\left(l\right)}G$$

(14) $${\tilde{S}}_{total}^{\left(l\right)}=\sum_{i=1}^{D}\sum_{j=1}^{N_{s}}\left(G^{H}Y_{ij}^{\left(l\right)}-G^{H}m_{total}^{\left(l\right)}\right){\left(G^{H}Y_{ij}^{\left(l\right)}-G^{H}m_{total}^{\left(l\right)}\right)}^{H}=G^{H}S_{total}^{\left(l\right)}G.$$

Note that it is desirable to maximize the trace of ${\tilde{S}}_{b}^{\left(l\right)}$ and simultaneously minimize the trace of ${\tilde{S}}_{w}^{\left(l\right)}$ for the motion recognition in the reduced dimensional space. Accordingly, the optimal linear transformation matrix G at the *l*th radar can be found thusly:

(15) $${\hat{G}}^{\left(l\right)}=\underset{G}{\arg\max}J^{\left(l\right)}\left(G\right),\;J^{\left(l\right)}\left(G\right)≜\frac{trace(G^{H}S_{b}^{\left(l\right)}G)}{trace(G^{H}S_{w}^{\left(l\right)}G)}.$$

From (5), the scatter matrix in (8) can be rewritten as

(16) $$\begin{array}{ccc} {S_{w}}^{\left(l\right)} & = & {\sum_{i=1}}^{D}{\sum_{j=1}}^{N_{s}}\left({\alpha_{i}}^{\left(l\right)}{F_{ij}}^{\left(l\right)}+{N_{ij}}^{\left(l\right)}-{m_{i}}^{\left(l\right)}\right){\left({\alpha_{i}}^{\left(l\right)}{F_{ij}}^{\left(l\right)}+{N_{ij}}^{\left(l\right)}-{m_{i}}^{\left(l\right)}\right)}^{H}\\ & = & {{|\alpha_{i}}^{\left(l\right)}|}^{2}{S_{w}^{\prime }}^{\left(l\right)}+{{\sigma_{n}}^{2}I}_{MN}, \end{array}$$

where ${S_{w}^{\prime }}^{\left(l\right)}$ is the within-cluster scatter matrix excluding the channel gain and the noise. We can also derive ${S_{b}}^{\left(l\right)}={|\alpha_{i}^{\left(l\right)}|}^{2}{S_{b}^{\prime }}^{\left(l\right)}$. Accordingly, (15) can be given as

(17) $${\hat{G}}^{\left(l\right)}=\underset{G}{\arg\max}\frac{trace(G^{H}{S_{b}^{\prime }}^{\left(l\right)}G)}{trace(G^{H}\left({S_{w}^{\prime }}^{\left(l\right)}+\sigma_{n}^{2}/{|\alpha_{i}^{\left(l\right)}|}^{2}I_{MN}\right)G)}.$$

We note that the optimal transformation ${\hat{G}}^{\left(l\right)}$ in (17) can be obtained by the generalized eigenvectors associated with the $M_{d}$ largest generalized eigenvalues of the matrix pair $\left({S_{b}^{\prime }}^{\left(l\right)},\;{S_{w}^{\prime }}^{\left(l\right)}+\sigma_{n}^{2}/|{\alpha_{i}^{\left(l\right)}|}^{2}I_{MN}\right)$. Furthermore, it can be efficiently computed through the GSVD algorithm [11,13,14], which is modified to our motion recognition scenario in Algorithm 1.
**Algorithm 1** LDA/GSVD algorithm for motion recognition:From (7)–(9), set $H_{b}$ and $H_{w}$ as:
(18)$$H_{b}=\sqrt{N_{s}}\left[m_{1}^{\left(l\right)}-m_{total}^{\left(l\right)},m_{2}^{\left(l\right)}-m_{total}^{\left(l\right)},\ldots ,m_{D}^{\left(l\right)}-m_{total}^{\left(l\right)}\right]$$
(19)$$H_{w}=\left[A_{1}^{\left(l\right)}-m_{1}^{\left(l\right)}1_{N_{s}}^{T},A_{2}^{\left(l\right)}-m_{2}^{\left(l\right)}1_{N_{s}}^{T},\ldots ,A_{D}^{\left(l\right)}-m_{D}^{\left(l\right)}1_{N_{s}}^{T}\right].$$Compute the SVD of $Z=\left[\begin{array}{c} H_{b}^{H}\\ H_{w}^{H} \end{array}\right]\in C^{D(N_{s}+1)\times MN}$. That is,
(20)$$\begin{array}{c} Z=P\left[\begin{array}{cc} \Lambda & 0\\ 0 & 0 \end{array}\right]U^{H}, \end{array}$$
where $P\in C^{D\left(N_{s}+1\right)\times D\left(N_{s}+1\right)}$ and $U\in C^{MN\times MN}$ are orthogonal and Λ is an s×s diagonal matrix. Here, *s* is an effective rank of Z.Partition the matrix P as
(21)$$\begin{array}{c} P=\left[\;\begin{array}{cc} P_{11} & P_{12}\\ P_{21} & P_{22} \end{array}\;\right], \end{array}$$
where $P_{11}\in C^{D\times s}$, $P_{12}\in C^{D\times (D\left(N_{s}+1\right)-s)}$, $P_{21}\in C^{DN_{s}\times s}$, and $P_{22}\in C^{DN_{s}\times \left(D\left(N_{s}+1\right)-s\right)}$ are submatrices of P; and compute the orthogonal matrix V from the SVD of $P_{11}$$\left(={W\Sigma V}^{H}\right)$.Compute X as
(22)$$\begin{array}{c} X=U\left[\begin{array}{cc} \Lambda^{-1}V & 0\\ 0 & I \end{array}\right]. \end{array}$$Then, set the transformation matrix ${\bar{G}}^{\left(l\right)}$ as
(23)$$\begin{array}{c} {\bar{G}}^{\left(l\right)}=\left[{\left[X\right]}_{1},{\left[X\right]}_{2},\ldots ,{\left[X\right]}_{M_{d}}\right]\in C^{MN\times M_{d}}, \end{array}$$
where ${\left[A\right]}_{i}$ denotes the *i*th column of a matrix A.

Once ${\bar{G}}^{\left(l\right)}$ is obtained in (23), we can transform the MD signature data in (4) into a lower dimensional space. Accordingly, the motion can be identified via

(24) $$\hat{i}=\underset{i}{\arg\min}z_{i}^{\left(l\right)},$$

where $z_{i}^{\left(l\right)}$ is denoted as the distance of each piece of MD signature data from the center of a cluster, given by

(25) $$z_{i}^{\left(l\right)}={∥{\bar{G}}^{(l)H}Y_{test}^{\left(l\right)}-{\tilde{m}}_{i}^{\left(l\right)}∥}_{2}^{2}.$$

Here, ${\tilde{m}}_{i}^{\left(l\right)}$ is the representative vector for the *i*th motion in the reduced dimensional space, given as ${\tilde{m}}_{i}^{\left(l\right)}={\bar{G}}^{(l)H}m_{i}^{\left(l\right)}$.

**Remark** **1.** *Note that, in (18) and (19), $S_{b}^{\left(l\right)}=H_{b}H_{b}^{H}$ and $S_{w}^{\left(l\right)}=H_{w}H_{w}^{H}$. In addition, from (20) and (21),*

(26) $$\begin{array}{c} \;\left[\begin{array}{c} H_{b}^{H}\\ H_{w}^{H} \end{array}\right]U\;=\;\left[\begin{array}{cc} P_{11}\Lambda & 0\\ P_{21}\Lambda & 0 \end{array}\right] \end{array}$$

*Then, from the SVD of $P_{11}$ and [11,19], we can have*

(27) $$\begin{array}{c} W^{H}H_{b}^{H}X=\left(\Sigma ,0\right),\;{\bar{W}}^{H}H_{w}^{H}X=\left(\bar{\Sigma },0\right), \end{array}$$

*where $P_{21}=\bar{W}\bar{\Sigma }V^{H}$. Equivalently,*

(28) $$\begin{array}{c} X^{H}H_{b}H_{b}^{H}X=\left[\begin{array}{cc} \Sigma^{H}\Sigma & 0\\ 0 & 0 \end{array}\right],\\ X^{H}H_{w}H_{w}^{H}X=\left[\begin{array}{cc} {\bar{\Sigma }}^{H}\bar{\Sigma } & 0\\ 0 & 0 \end{array}\right]. \end{array}$$

*Therefore, the columns of X are the generalized eigenvalues of the matrix pair $\left({S_{b}^{\prime }}^{\left(l\right)},\;{S_{w}^{\prime }}^{\left(l\right)}+\sigma_{n}^{2}/|\alpha_{i}^{\left(l\right)}{|}^{2}I_{MN}\right)$.* □

**Remark** **2.** *The cost function $J^{\left(l\right)}\left({\hat{G}}^{\left(l\right)}\right)$ in (15) is denoted as the separability. That is, when $J^{\left(l\right)}\left({\hat{G}}^{\left(l\right)}\right)$ is larger, the dimensionally-reduced sample data vectors are well clustered and the motion recognition performance is more improved. Furthermore, from (17), the separability is proportional to the value of $\frac{|\alpha_{i}^{\left(l\right)}{|}^{2}}{\sigma_{n}^{2}}$, which implies that the received SNR at each radar affects the motion recognition.* □

## 4. Motion Classification at the Data Fusion Center

As the MD signature generated from the radar echo signal is heavily dependent on the aspect angle of the target relative to the radar’s line of sight, the multi-aspect MD signature data can be exploited for the motion classification. However, when the dimensionailty of the MD signature data is large, the burden on the backhaul link increases. Accordingly, in this section, we describe how the dimensionally-reduced data is reported to the data fusion center effectively and how the reported data can be processed for the motion classification at the data fusion center.

### 4.1. Strategy 1: Motion Classification at the Data Fusion Center with the Dimensionally-Reduced Data

In Section 3, we can effectively reduce the dimension of the MD signature data while maintaining the separability by using the GSVD/LDA algorithm. Accordingly, the *l*th radar can transmit ${\bar{G}}^{(l)H}Y^{\left(l\right)}$ to the data fusion center, rather than transmitting the MD signature $Y^{\left(l\right)}$.

Specifically, by exploiting the collected datasets in (7), the aggregated datasets can be formulated as

(29) $$A=\left[\begin{array}{ccc} {G^{\left(1\right)}}^{H}{A_{1}}^{\left(1\right)} & \ldots & {G^{\left(1\right)}}^{H}{A_{D}}^{\left(1\right)}\\ {G^{\left(2\right)}}^{H}{A_{1}}^{\left(2\right)} & \ldots & {G^{\left(2\right)}}^{H}{A_{D}}^{\left(2\right)}\\ \vdots & \ddots & \vdots \\ {G^{\left(L\right)}}^{H}{A_{1}}^{\left(L\right)} & \ldots & {G^{\left(L\right)}}^{H}{A_{D}}^{\left(L\right)} \end{array}\right]=\left[\begin{array}{ccc} A_{1} & \ldots & A_{D} \end{array}\right]\in C^{LM_{d}\times DN_{s}}.$$

Again, the average of the samples in the *i*th cluster, $m_{i}$, can be computed at the data fusion center as $m_{i}=\frac{1}{N_{s}}\sum_{j=1}^{N_{s}}{\left[A_{i}\right]}_{j}$, and it can be denoted as the representative vector for the *i*th motion at the data fusion center. Therefore, when ${\bar{G}}^{(l)H}Y_{test}^{\left(l\right)}$ is received from the *l*th radar for a certain motion, $Y_{test}$ is formulated as

(30) $$Y_{test}=\left[\begin{array}{c} {\bar{G}}^{(1)H}Y_{test}^{\left(1\right)}\\ {\bar{G}}^{(2)H}Y_{test}^{\left(2\right)}\\ \vdots \\ {\bar{G}}^{(L)H}Y_{test}^{\left(L\right)} \end{array}\right],$$

and the motion can be identified such as

(31) $$\hat{i}=\underset{i}{\arg\min}{∥Y_{test}-m_{i}∥}_{2}^{2}.$$

We note that the dimension of $Y_{test}$ can be further reduced through the linear transformation matrix $G\in C^{LM_{d}\times M_{d}}$, which can be found by applying the GSVD/LDA algorithm to (29).

### 4.2. Strategy 2: Motion Classification at the Data Fusion Center with Softmax-Processed Data

Even though the dimensions of the MD signature data are reduced via strategy 1, the elements of $G^{(l)H}A_{d}^{\left(l\right)}$ have continuous complex values, which should be properly quantized when the backhaul link has a limited capacity. From (6), $N_{l}$ bits can be transmitted error-freely for single-channel use. A naive element-wise quantization incurs a significant distortion on the dimensionally-reduced MD signature data. Note that from (24), the cluster index of the data is determined to have the shortest distance from the center (i.e., ${\tilde{m}}_{i}^{\left(l\right)}$) of each cluster. Accordingly, rather than transmitting the dimensionally-reduced MD signature data, by reporting the distances from the centers of clusters (i.e., $z_{i}^{\left(l\right)}$ in (25)), each radar can deliver useful information for cluster selection with limited resources.

To transmit $z_{i}^{\left(l\right)}$ to the data center for the motion classification, we use the softmax process, which is widely applied to various multiclass classification problems, such as multinomial logistic regression and multiclass linear discriminant analysis [20]. The output of the softmax process at the *l*th radar is then given by

(32) $$P_{i}^{\left(l\right)}=\frac{\exp∥z_{i}^{\left(l\right)}∥}{\exp∥z_{1}^{\left(l\right)}∥+\exp∥z_{2}^{\left(l\right)}∥+\cdots +\exp∥z_{D}^{\left(l\right)}∥},$$

for i=1,…,D. At the fusion center, the motion can be identified such as

(33) $$\hat{i}=\underset{i}{\arg\max}\sum_{l=1}^{L}P_{i}^{\left(l\right)}.$$

To improve the classification performance, because the cost function $J^{\left(l\right)}\left(G\right)$ indicates separability at the *l*th radar from Remark 2, the motion can be identified by maximizing the separability-weighted output of the softmax process:

(34) $$\hat{i}=\underset{i}{\arg\max}\sum_{l=1}^{L}w_{l}P_{i}^{\left(l\right)},$$

where $w_{l}=\frac{J^{\left(l\right)}\left({\hat{G}}^{\left(l\right)}\right)}{\sum_{l=1}^{L}J^{\left(l\right)}\left({\hat{G}}^{\left(l\right)}\right)}$.

### 4.3. Pyramid Vector Quantization and Bit Allocation for a Limited Backhaul Link

#### 4.3.1. Pyramid Vector Quantization for Limited Backhaul Link

Since $\sum_{i}^{D}P_{i}^{\left(l\right)}=1$ from (32), PVQ can be applied to transmit

(35) $$P^{\left(l\right)}={\left[P_{1}^{\left(l\right)},\ldots ,P_{D}^{\left(l\right)}\right]}^{T},$$

through the limited backhaul link with $N_{l}$ bit per channel use. We note that PVQ is an efficient quantization method when the sum of vector components is fixed as a constant, where the codebook of PVQ consists of cubic lattice points on the *L*-dimensional pyramid [15]. That is, the components of *L*-dimensional vector on pyramid are integer-valued and the sum of total components is fixed as an integer, *K*. Accordingly, by denoting N(L,K) as the set of codewords in the PVQ codebook, it is given as

(36) $$N\left(L,K\right)=\left\{c_{p}≜\left(x_{1}^{p},\ldots ,x_{L}^{p}\right)\;|\;\sum_{i=1}^{L}x_{i}^{p}=K,\;x_{i}^{p}\in Z^{+}\cup \left\{0\right\}\right\}.$$

Throughout the paper, considering quantization of the softmax output in (32), we assume that $x_{i}$ are non-negative integers in (36). Then, the number of codewords in N(L,K), P(L,K), can be computed as Cadel and Parladori [16]

(37) P(L,K)=L+K−1K,

or

**Figure 2:**
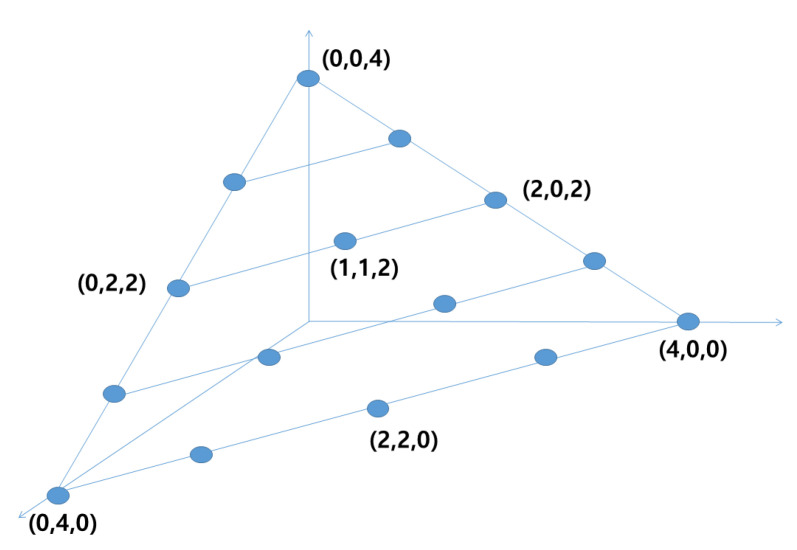
Codebook of pyramid vector quantization, L=3,K=4.

(38) P(L,K)=P(L−1,K)+P(L,K−1),

where ab is the binomial coefficient given as $=\frac{a!}{b!(a-b)!}$. Then, the number of required bits to transmit the codewords in N(L,K) is given as $\left\lceil \log_{2}P\left(L,K\right)\right\rceil$, where ⌈·⌉ is the ceiling operation. For example, the codebook of N(3,4) is shown in Figure 2, where the codewords are in the three dimensional space and the total number of codewords is given as P(3,4)=15. Accordingly, four bits are required to transmit the codeword per channel use.

To exploit the PVQ to quantize $P^{\left(l\right)}$ in (35), the normalized PVQ codebook is defined as

(39) $$\bar{N}\left(L,K\right)=\left\{{\bar{c}}_{p}≜\left(\left.\frac{x_{1}^{p}}{K},...,\frac{x_{L}^{p}}{K}\right)\;\right|\;\sum_{i=1}^{L}\frac{x_{i}^{p}}{K}=1,\;x_{i}^{p}\in Z^{+}\cup \left\{0\right\}\right\}.$$

**Figure 3:**
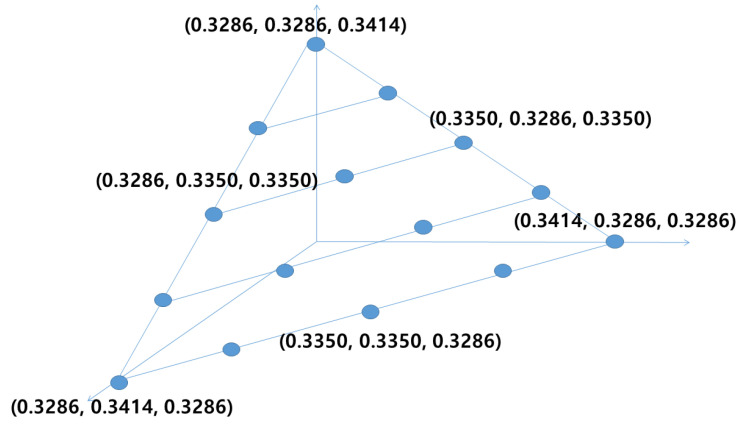
Normalized codebook of pyramid vector quantization, L=3,K=4.

Figure 3 shows an example of the normalized codebook (3, 4).

When the output of the softmax process at the *l*th radar is given as $P^{\left(l\right)}$ and the $N_{l}$ bits can be transmitted per channel use of the backhaul link, from (37), we can design $\bar{N}\left(D,K^{l}\right)$, where $K^{l}$ is determined as the maximum *K* that satisfies the condition of $P\left(D,K\right)\leq 2^{N_{l}}$. Then, $P^{\left(l\right)}$ can be quantized as

(40) $${\hat{\bar{c}}}^{\left(l\right)}=\underset{{\bar{c}}_{p}\in \bar{N}\left(D,K^{l}\right)}{\arg\min}{∥P^{\left(l\right)}-{\bar{c}}_{p}∥}^{2}$$

which can be forwarded to the data fusion center. Then, at the data fusion center, the motion can be identified by maximizing the separability-weighted output of the softmax process:

(41) $$\hat{i}=\underset{i}{\arg\max}\sum_{l=1}^{L}w_{l}{\left[{\hat{\bar{c}}}^{\left(l\right)}\right]}_{i},$$

which is analogous to (34).

#### 4.3.2. Bit allocation for Limited Backhaul Link

From (37) and (39), it can be found that, as the number of bits increases, the associated *K* can be increased. That is, the Euclidean distance between the codewords is reduced, resulting in the reduction of quantization errors. Accordingly, by allocating more bits (i.e., more resources of backhaul link) to the distributed radar with larger separability, the motion recognition performance at the data fusion center can be further improved. Specifically, from (6), the number of bits per channel use for the *l*th radar, $N_{l}$, is determined as

(42) $$N_{l}=\left\lceil w_{l}N_{total}\right\rfloor ,$$

where $w_{l}$ is defined in (34) and ⌈·⌋ is the rounding operation. By allocating more resources of backhaul link to the radar with a larger separability value, the data from that radar can be exploited at the data fusion center with smaller quantization error.

Interestingly, from Remark 2 and (17), when the separability excluding the channel gain and the noise (i.e., the ratio of the traces of two matrices, $S_{b}^{{}^{\prime }\left(l\right)}$ and $S_{w}^{{}^{\prime }\left(l\right)}$) are the same for all the radars, the radar with higher received SNR can be allocated more backhaul link resources.

## 5. Simulation and Experiment Results

### 5.1. Simulation with MNIST Hand-Writing Data

Before implementing the motion classification using distributed MD radars with limited backhaul, we first verified the classification performance of the GSVD/LDA-based dimension reduction in a distributed system by applying it to the image classification problem with MNIST hand-writing data [17]. We note that the MNIST dataset consists of hand-written numbers from 0 to 9; it is widely used as pilot data for image classification, including the deep learning system [21]. Specifically, in this subsection, the classification of the number set {1,2,3} from the hand-written images with 784 pixels is considered. Accordingly, the hand-written images with 784 pixels were exploited instead of the MD signature $F_{ij}^{\left(l\right)}$ in (5).

**Figure 4:**
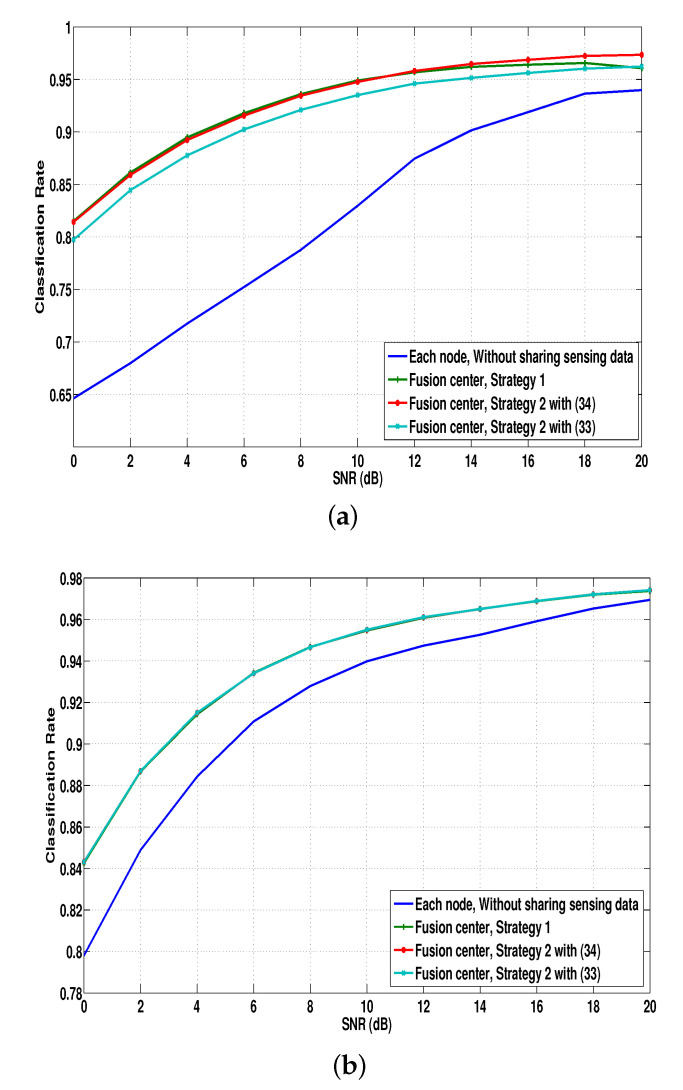
Average recognition rates for (**a**) $\left[\alpha^{\left(1\right)},\alpha^{\left(2\right)},\alpha^{\left(3\right)}\right]=\left[0.14,1,1.4\right]$ and (**b**) $\left[\alpha^{\left(1\right)},\alpha^{\left(2\right)},\alpha^{\left(3\right)}\right]=\left[1,1,1\right]$.

Throughout the simulation, the number of nodes was set to L=3, and at each node, $G^{\left(l\right)}$ was computed by exploiting 1000 MNIST training data for each number. In Figure 4, the average classification rates are provided for strategy 1 in Section 4.1 and strategy 2 with (33) and (34) in Section 4.2 when (a) $\left[\alpha^{\left(1\right)},\alpha^{\left(2\right)},\alpha^{\left(3\right)}\right]=\left[0.14,1,1.4\right]$ and (b) $\left[\alpha^{\left(1\right)},\alpha^{\left(2\right)},\alpha^{\left(3\right)}\right]=\left[1,1,1\right]$. For comparison purposes, we also evaluate the averages of the classification rates at distributed nodes without sharing their sensing data to the fusion center. The quantization of the sharing data from each node to the fusion center through the backhaul link was not considered.

As shown in Figure 4a, it is obvious that the classification rates at the fusion center are better than those of each node without sharing the sensing data. Interestingly, strategy 2 with (34) (i.e., using the separability-weighted output of the softmax process) outperformed other schemes, because the path-loss (or SNR) affects the separability at each node, as discussed in Remark 2, and the classification quality at each node can be reflected on the final classification decision when strategy 2 with (34) is exploited. In Figure 4b with the same path loss, the performances of strategy 2 with (33) and (34) are almost the same.

**Figure 5:**
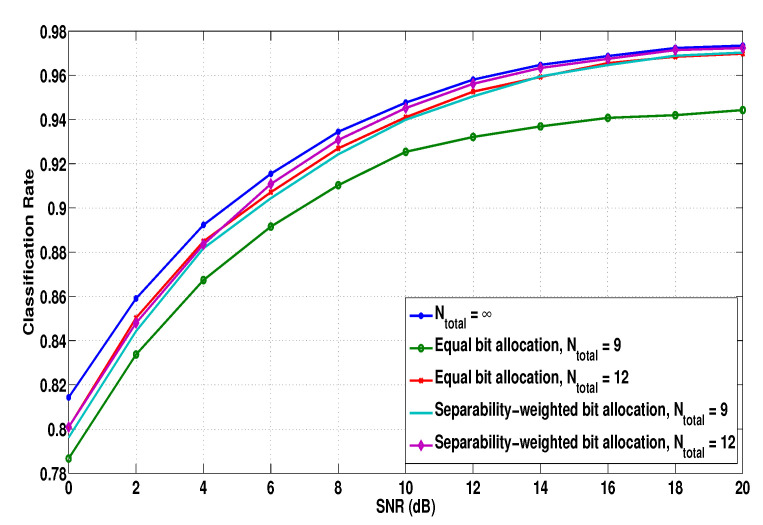
Average recognition rates when PVQ is applied and the path-loss components experienced by each node’s signal are significantly different.

To validate the proposed PVQ with the bit allocation for limited backhaul link, we evaluate the classification rates for $N_{total}=\left\{9,12,\infty \right\}$ and $\left[\alpha^{\left(1\right)},\alpha^{\left(2\right)},\alpha^{\left(3\right)}\right]=\left[0.14,1,1.4\right]$ in Figure 5.

The case of $N_{total}=\infty$ implies that the backhaul link does not have any resource limitation and the sharing data from each node are unquantized and reported to the fusion center through the backhaul link without any distortion. From the figure, it can be found that, as $N_{total}$ increases, the classification performance improves. In addition, when the resources are allocated proportionally to the separability, as in (42), the classification performance can be further improved compared to that with equal resource allocation over the distributed nodes.

### 5.2. A Test with MD Signatures Measured through USRP-Implemented Radars

**Figure 6:**
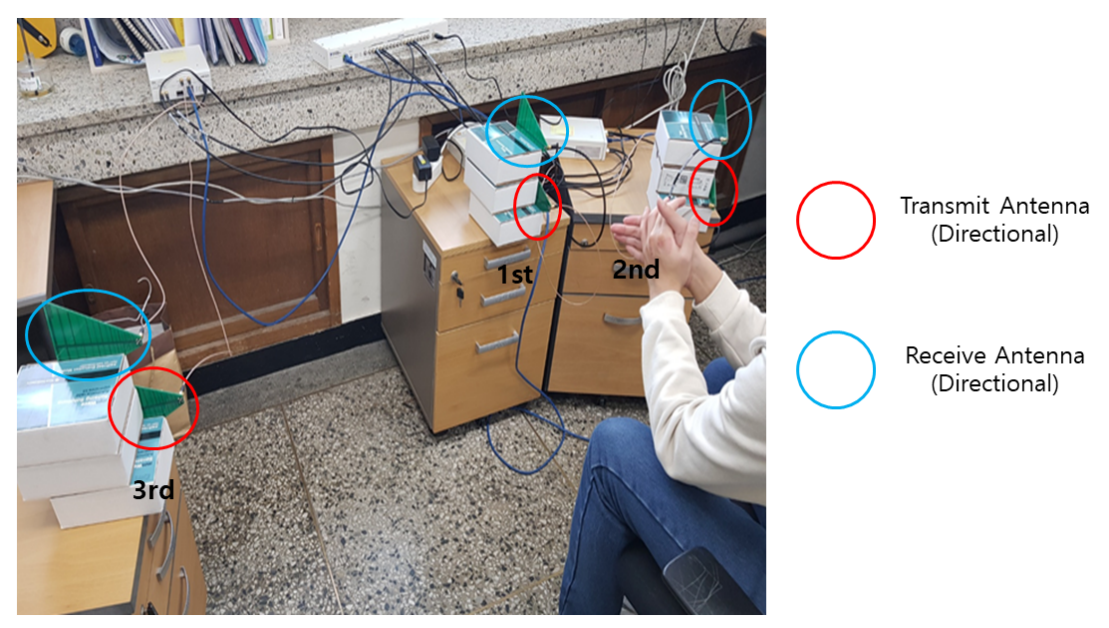
Experiment setting for three distributed radars.

**Figure 7:**
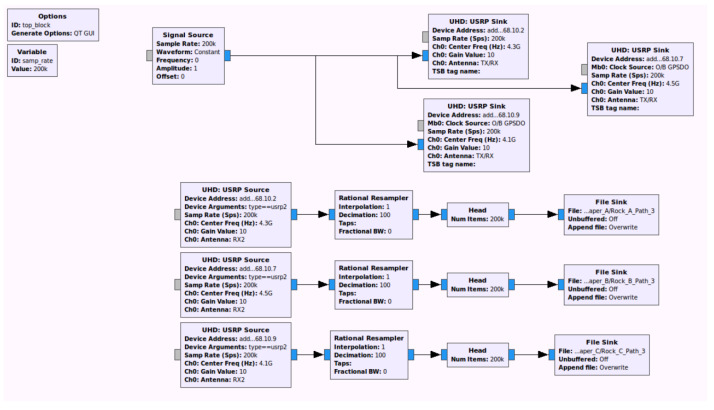
GNU-radio flowgraph for distributed MD radars.

To verify the performance of the proposed LDA-based motion classification using the distributed MD radars, we have implemented the distributed MD radars by exploiting the USRP N210 devices with CBX daughterboards and log periodic PCB directional antennas [18], as in Figure 6, and the associated GNU-radio flowgraph is shown in Figure 7. Here, the carrier frequencies for three MD radars were set as $\left[f_{c}^{\left(1\right)},f_{c}^{\left(2\right)},f_{c}^{\left(3\right)}\right]=\left[4.1,4.3,4.5\right]$ GHz, and the sampling rate was set as 200 kHz. Note that we exploited the multi-frequencies with a large difference (i.e., 200 MHz) to avoid inter-radar interference without increasing the implementation complexity, but the frequency difference can be further reduced if the proper resource scheduling method is exploited. In addition, the center carrier frequency of 4.3 GHz was exploited as used in Liu and Chen [22] for the hand motion-aware radar, but the proposed scheme can be extended to other frequency bands without difficulty. Throughout the experiment, the number of time samples and the window size for the STFT were set as N=2533 and M=63.

**Figure 8:**
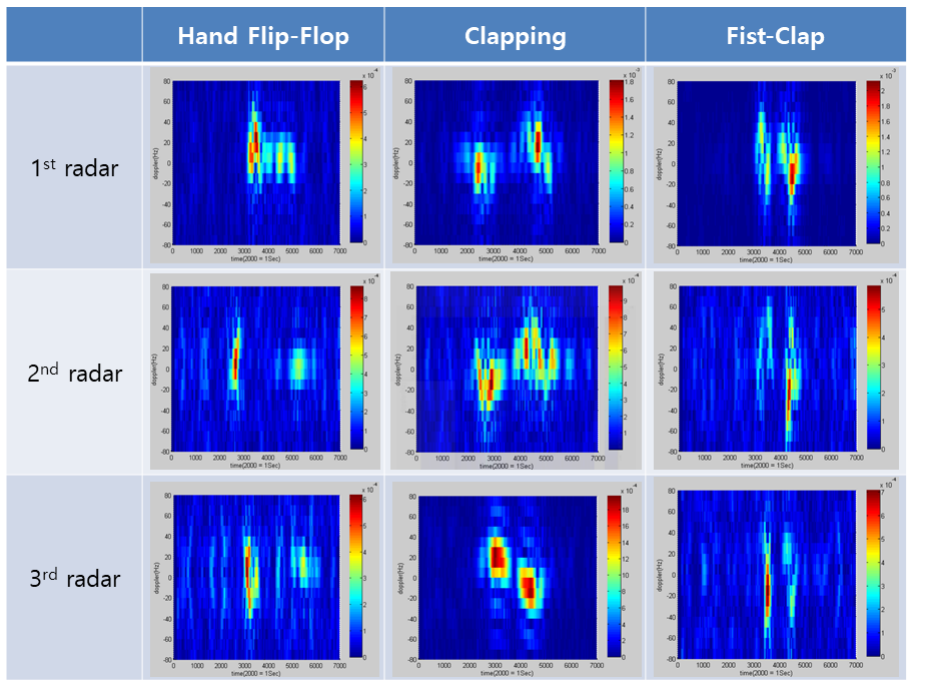
MD signatures of three different hand gestures at distributed radars.

For three different hand gestures—hand flip-flop, clapping, and fist-clap—the MD signatures were collected by exploiting the distributed MD radars, and the associated snapshots are provided in Figure 8. We note that the aspect angles of the same gesture were different for distributed radars, and accordingly, the MD signatures appeared differently at distributed radars, even for the same gesture.

In Table 1, the motion classification rates are listed for the experimental settings in Figure 6. Here, 120 MD signature samples per motion were collected in each radar, and half of these were randomly chosen and exploited to compute $G^{\left(l\right)}\left(l=1,2,3\right)$, and the other 60 samples were used to test the classification performance. In Table 1, motions 1, 2, and 3 correspond to hand flip-flop, clapping, and fist-clap, respectively. As for the experimental results without the proposed strategies, we evaluated the classification performance at a single radar without data sharing to the fusion center. In addition, we evaluated the performance for the scenario in which the backhaul link between the distributed radar and fusion center was wired, implying that $N_{total}=\infty$, denoting strategy 1 with $N_{total}=\infty$.

We note that the separabilities of the distributed radars are given as [0.08740.06330.1183], which implies that the MD signatures from the third radar are more beneficial for the classification. From the Table 1, it can be found that combining multi-aspect angles from distributed radars at the fusion center outperforms the classification at a single radar without data sharing to the fusion center. In addition, as the number of bits per channel use (i.e., the resource of the backhaul link) increases, the classification rate also increases. Importantly, when the resources are allocated proportionally to the separability, as in (42), the classification rate can be further improved compared to that with equal bit allocation over the distributed nodes, which coincides with the observation in the simulation with MNIST hand-writing data.

**Figure 9:**
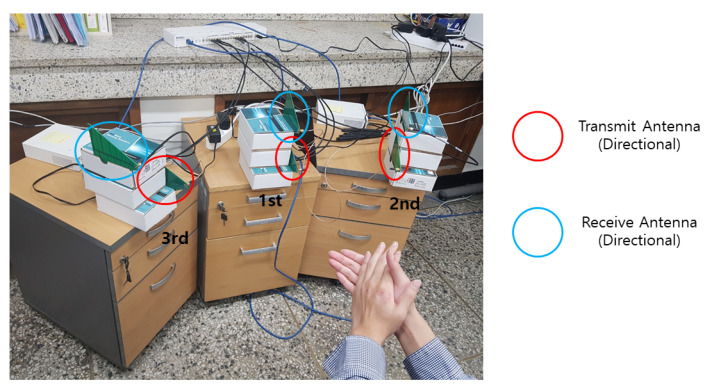
Experimental settings for the distributed radars which were placed close together.

To see the effect of the diversity of the multi-aspect angles on the recognition performance, we performed an additional motion classification experiment when the distributed radars were placed close together, as in Figure 9. Table 2 shows the classification rates for the experiment from Figure 9. The separabilities of distributed radars were [0.04260.12120.1272]. We can find a similar trend in Table 1, where combining multi-aspect angles at the fusion center outperformed the classification at a single radar, and when the resources were allocated proportionally to the separability, the classification rate could be further improved. Interestingly, the overall classification rates in Table 2 are worse than those in Table 1, because the diversity of the multi-aspect MD signature data is reduced when the distributed radars are placed close together. Specifically, when the distributed radars are placed close together, as in Figure 9, the radar signal received by each radar has a similar incoming angle from the target. In contrast, in Figure 6, the third radar can observe the target motion with a relatively different aspect angle compared to other distributed radars. Accordingly, the diversity of the multi-aspect MD signature data is decreased when the distributed radars are placed close together as in Figure 9; compare to Figure 6. This resulted in the degradation of the overall classification rates in Table 2 compared to those in Table 1. When the radars were co-located, the recognition rate of the proposed strategies was low, especially for motion 3, because motion 3 (fist-clap) had a similar MD signature to other actions. Note that this performance degradation can be overcome by increasing the diversity of multi-aspect angle as in Figure 6.

## 6. Conclusions

In this paper, we proposed an LDA-based motion classification algorithm using the MD signatures obtained from distributed MD radars, in which the the distributed radars are connected to the data fusion center through the limited backhaul link. Due to the limited backhaul link, at each radar, the dimensions of MD signature are reduced by using the LDA algorithm, and the dimensionally-reduced MD signatures from multiple perspectives can be collected at the data fusion center. To further reduce the burden of the backhaul link, we also propose the softmax processing method and that the output of the softmax process at each radar should be quantized through the PVQ with a finite number of bits and is reported to the data fusion center. To improve the classification performance, the channel resources of the backhaul link are adaptively allocated based on the classification separability at each radar. Through computer simulations and an experiment, we demonstrated that the proposed algorithm (i.e., LDA-based motion classification with softmax processing, PVQ, and the separability-weighted bit allocation) exhibits a considerable performance improvement in the limited backhaul link, which is comparable to that without any resource limitation in the backhaul link.

## Figures and Tables

**Table 1:** Classification rates for the experiment settings in Figure 6.

	Motion 1	Motion 2	Motion 3	Total Mean
Average of distributed Radars without data sharing to fusion center	0.7222	0.9278	0.8944	0.8481
Strategy 1 with $N_{total}=\infty$	1.0000	0.9500	0.7167	0.8889
Strategy 2 with $N_{total}=9$ Equal bit allocation	1.0000	0.9333	0.7500	0.8944
Strategy 2 with $N_{total}=9$ Separability-weighted bit allocation	1.0000	0.9167	0.8833	0.9333
Strategy 2 with $N_{total}=12$ Equal bit allocation	1.0000	0.9167	0.7833	0.9
Strategy 2 with $N_{total}=12$ Separability-weighted bit allocation	1.0000	0.9333	0.9833	0.9722

**Table 2:** Classification rates for the experiment in Figure 9.

	Motion 1	Motion 2	Motion 3	Total Mean
Average of distributed Radars without sharing to Fusion center	0.7722	0.7677	0.8000	0.7800
Strategy 1 with $N_{total}=\infty$	1.0000	0.9667	0.8167	0.9278
Strategy 2 with $N_{total}=9$ Equal bit allocation	0.9667	0.9000	0.7333	0.8667
Strategy 2 with $N_{total}=9$ Separability-weighted bit allocation	1.0000	0.9000	0.7167	0.8722
Strategy 2 with $N_{total}=12$ Equal bit allocation	0.9833	0.9167	0.7000	0.8667
Strategy 2 with $N_{total}=12$ Separability-weighted bit allocation	0.9667	0.9167	0.8333	0.9056

## Data Availability

The data presented in this study are available on request from the corresponding author. The data are not publicly available due to confidentiality reasons.

## References

[1] Li G., Zhang R., Ritchie M., Griffiths H. (2018). Sparsity-Driven Micro-Doppler Feature Extraction for Dynamic Hand Gesture Recognition. IEEE Trans. Aerosp. Electron. Syst..

[2] Wan Q., Li Y., Li C., Pal R. Gesture recognition for smart home applications using portable radar sensors. Proceedings of the 2014 36th Annual International Conference of the IEEE Engineering in Medicine and Biology Society.

[3] Sakamoto T., Gao X., Yavari E., Rahman A., Boric-Lubecke O., Lubecke V.M. (2018). Hand Gesture Recognition Using a Radar Echo I? Plot and a Convolutional Neural Network. IEEE Sens. Lett..

[4] Amin M.G., Zeng Z., Shan T. Hand Gesture Recognition based on Radar Micro-Doppler Signature Envelopes. Proceedings of the 2019 IEEE Radar Conference (RadarConf).

[5] Hoffmann F., Ritchie M., Fioranelli F., Charlish A., Griffiths H. Micro-Doppler based detection and tracking of UAVs with multistatic radar. Proceedings of the 2016 IEEE Radar Conference (RadarConf).

[6] Fioranelli F., Ritchie M., Griffiths H. (2015). Aspect angle dependence and multistatic data fusion for micro-Doppler classification of armed/unarmed personnel. IET Radar Sonar Navig..

[7] Vespe M., Baker C.J., Griffiths H.D. (2007). Radar target classification using multiple perspectives. IET Radar Sonar Navig..

[8] Özcan M.B., Gürbüz S.Z., Persico A.R., Clemente C., Soraghan J. Performance analysis of co-located and distributed MIMO radar for micro-Doppler classification. Proceedings of the 2016 European Radar Conference (EuRAD).

[9] Smith G., Woodbridge K., Baker C., Griffiths H. (2010). Multistatic micro-Doppler radar signatures of personnel targets. Signal Process. IET.

[10] Fioranelli F., Ritchie M., Griffiths H. (2015). Classification of Unarmed/Armed Personnel Using the NetRAD Multistatic Radar for Micro-Doppler and Singular Value Decomposition Features. IEEE Geosci. Remote Sens. Lett..

[11] Howland P., Jeon M., Park H. (2003). Structure Preserving Dimension Reduction for Clustered Text Data Based on the Generalized Singular Value Decomposition. SIAM J. Matrix Anal. Appl..

[12] Ye J., Janardan R., Park C.H., Park H. (2004). An optimization criterion for generalized discriminant analysis on undersampled problems. IEEE Trans. Pattern Anal. Mach. Intell..

[13] Park C., Park H. (2008). A Comparison of Generalized Linear Discriminant Analysis Algorithms. Pattern Recogn..

[14] Park J., Chun J., Park H. (2010). Generalised singular value decomposition based algorithm for multi-user multiple-input multiple-output linear precoding and antenna selection. IET Commun..

[15] Fischer T. (1986). A pyramid vector quantizer. IEEE Trans. Inf. Theory.

[16] Cadel D., Parladori G. Pyramid vector coding for high quality audio compression. Proceedings of the 1997 IEEE International Conference on Acoustics, Speech, and Signal Processing.

[17] LeCun Y., Cortes C. MNIST Handwritten Digit Database-2010. http://yann.lecun.com/exdb/mnist/.

[18] USRP N210. https://www.ettus.com/all-products/un210-kit/.

[19] Paige C., Saunders M. (1981). Towards a Generalized Singular Value Decomposition. SIAM J. Numer. Anal..

[20] Bishop C.M. (2006). Pattern Recognition and Machine Learning.

[21] Srivastava R.K., Greff K., Schmidhuber J. (2015). Training very deep networks. arXiv.

[22] Liu B., Chen R. Software-defined radar and waveforms for studying micro-Doppler signatures. Proceedings of the Radar Sensor Technology XVIII. International Society for Optics and Photonics.

